# P-1864. Outpatient Parenteral Antimicrobial Therapy (OPAT) Practice Survey Updates 2025

**DOI:** 10.1093/ofid/ofaf695.2033

**Published:** 2026-01-11

**Authors:** Monica V Mahoney, Christina G Rivera (O'Connor), Laila M Castellino, Susan E Beekmann, Philip M Polgreen, Sara C Keller

**Affiliations:** Beth Israel Deaconess Medical Center, Boston, MA; Mayo Clinic, Rochester, MN; University of Texas Southwestern Medical Center, Dallas, TX; University of Iowa, IOWA CITY, Iowa; University of Iowa Carver College of Medicine, Iowa City, IA; Johns Hopkins University School of Medicine, Baltimore, MD

## Abstract

**Background:**

Outpatient parenteral antimicrobial therapy (OPAT) practice has continued to increase. We sought to understand current OPAT practices in a survey of U. S. clinicians.

**Figure 1:**
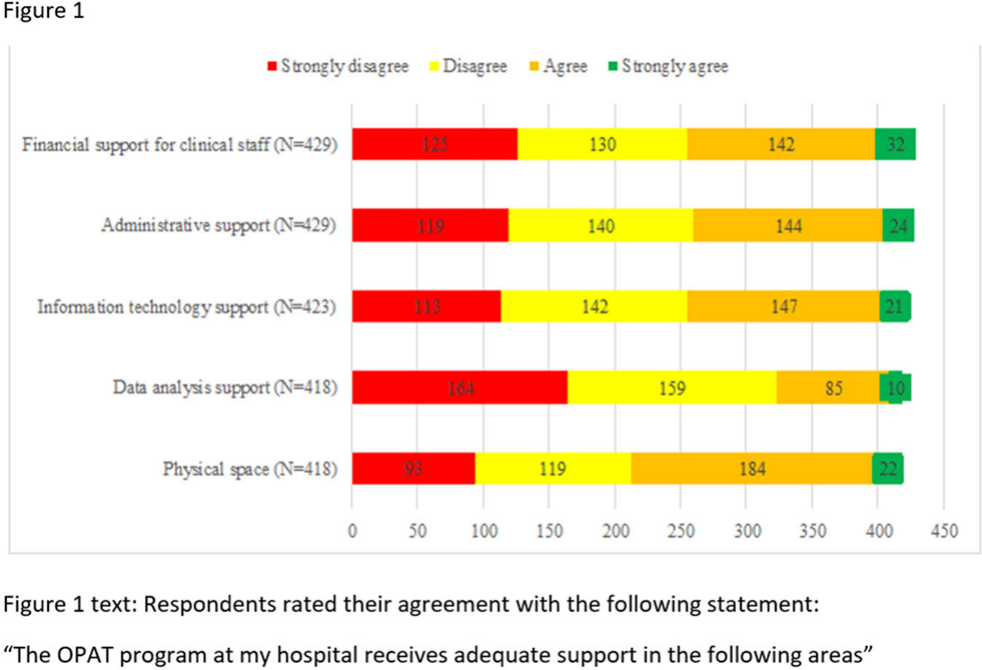
Respondents rated their agreement with the following statement: “The OPAT program at my hospital receives adequate support in the following areas”

**Figure 2:**
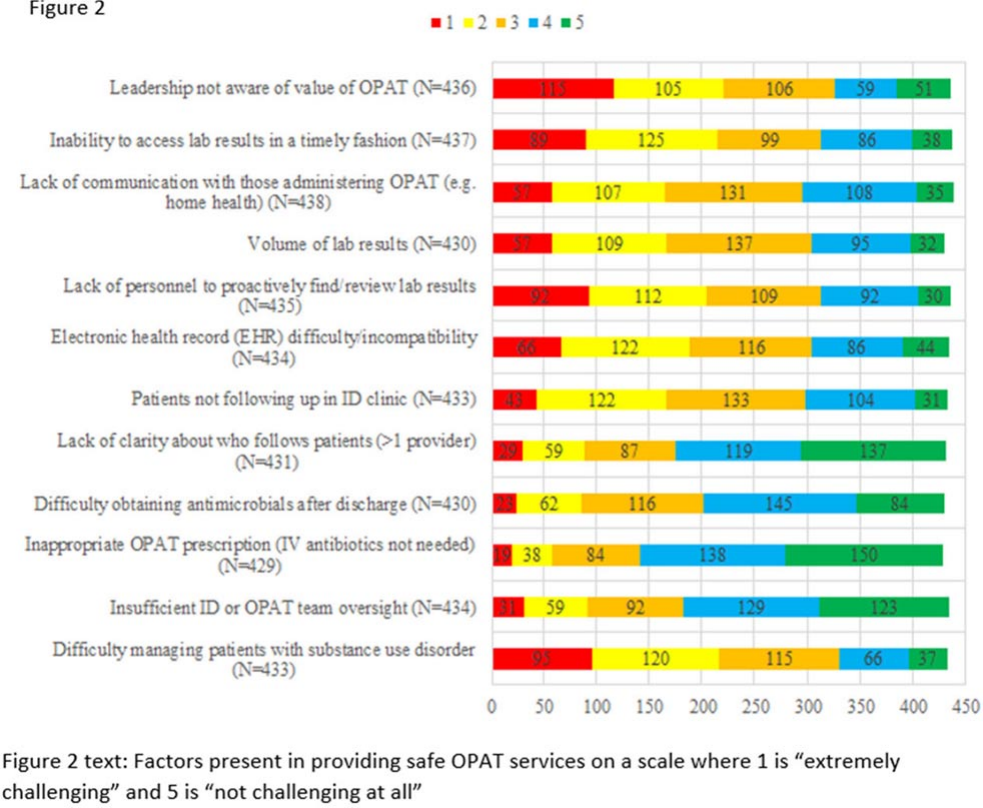
Factors present in providing safe OPAT services on a scale where 1 is “extremely challenging” and 5 is “not challenging at all”

**Methods:**

A survey instrument was developed by ID physicians, pharmacists and Infectious Diseases Society of America (IDSA) Emerging Infections Network (EIN) staff, utilizing prior EIN OPAT surveys. The survey focused on respondents’ role, responsibilities, time devoted to OPAT, structure of OPAT provision, location where OPAT is received, institutional support and oversight, role of complex outpatient antimicrobial therapy (COpAT) including oral antimicrobials and long-acting injectable agents, and barriers to safe OPAT care. The confidential survey was distributed to adult EIN members from Feb to March 2025.

**Results:**

Overall, 622 (38%) EIN members responded to the survey, of whom 469 with a role in managing OPAT patients were eligible to complete the survey. OPAT teams varied across programs with nearly all involving ID physicians (98%), majority involving ID/OPAT pharmacists (61%), nurses (60%), and nearly half involving administrative staff (48%) and advanced practice providers (APPs, 47%). ID consultation for OPAT enrollment was required in 60% of programs. Many programs (59%) report including COpAT. Response to outpatient lab results was largely managed by ID physicians (75%); however, multidisciplinary OPAT teams (37%), pharmacists (33%), and inpatient ID physicians (30%) were also frequently involved. Lab values were most often available via fax/email (43%) or electronic health record (EHR, 41%). Nearly 25% of programs “often/always” required proactive outreach to obtain safety labs.

Overall, many programs lacked data analysis, administrative, information technology, and financial support (Figure 1). Challenges faced by OPAT programs included lack of leadership awareness of the value of OPAT (51%), difficulty managing patients with substance use disorder (50%), access to timely lab values (48%), personnel to retrieve missing lab values (46%), and struggles with the EHR (43%) (Figure 2).

**Conclusion:**

Compared to EIN OPAT surveys in 2013 and 2019, programs have grown in size and composition, and expanded to COpAT. Similar problems persist regarding perceived lack of support.

**Disclosures:**

Monica V. Mahoney, PharmD, BCPS, BCIDP, FCCP, FIDSA, FIDP, FMSHP, gsk: Advisor/Consultant|seqirus: Advisor/Consultant Philip M. Polgreen, MD, Eli Lily: Advisor/Consultant|Pfizer: Grant/Research Support Sara C. Keller, MD, MPH, MSPH, CorMedix: Grant/Research Support

